# Chemical and Antimicrobial Characterization of *Mentha piperita* L. and *Rosmarinus officinalis* L. Essential Oils and In Vitro Potential Cytotoxic Effect in Human Colorectal Carcinoma Cells

**DOI:** 10.3390/molecules27186106

**Published:** 2022-09-19

**Authors:** Alina Dolghi, Dorina Coricovac, Stefania Dinu, Iulia Pinzaru, Cristina Adriana Dehelean, Cristina Grosu, Doina Chioran, Petru Eugen Merghes, Cristian Andrei Sarau

**Affiliations:** 1 Faculty of Pharmacy, “Victor Babes” University of Medicine and Pharmacy Timisoara, Eftimie Murgu Square No. 2, 300041 Timisoara, Romania; 2 Research Center for Pharmaco-Toxicological Evaluations, Faculty of Pharmacy, “Victor Babes” University of Medicine and Pharmacy Timisoara, Eftimie Murgu Square No. 2, 300041 Timisoara, Romania; 3 Faculty of Dental Medicine, “Victor Babes” University of Medicine and Pharmacy Timisoara, Eftimie Murgu Square No. 2, 300041 Timisoara, Romania; 4 Faculty of Bioengineering of Animal Resources, University of Life Science “King Michael I of Romania” from Timisoara, Calea Aradului 119, 300645 Timisoara, Romania; 5 Faculty of Medicine, “Victor Babes” University of Medicine and Pharmacy Timisoara, Eftimie Murgu Square No. 2, 300041 Timisoara, Romania

**Keywords:** *Mentha piperita* L. essential oil, *Rosmarinus officinalis* L. essential oil, colorectal cancer, cytotoxicity, antimicrobial potential

## Abstract

Colorectal cancer is one of the most frequently diagnosed forms of cancer, and the therapeutic solutions are frequently aggressive requiring improvements. Essential oils (EOs) are secondary metabolites of aromatic plants with important pharmacological properties that proved to be beneficial in multiple pathologies including cancer. *Mentha piperita* L. (M_EO) and *Rosmarinus officinalis* L. (R_EO) essential oils are well-known for their biological effects (antimicrobial, antioxidant, anti-inflammatory and cytotoxic in different cancer cells), but their potential as complementary treatment in colorectal cancer is underexplored. The aim of the present study was to investigate the M_EO and R_EO in terms of chemical composition, antioxidant, antimicrobial, and cytotoxic effects in a colorectal cancer cell line—HCT 116. The gas-chromatographic analysis revealed menthone and menthol, and eucalyptol, α-pinene and L-camphor as major compounds in M_EO and R_EO respectively. M_EO exhibited potent antimicrobial activity, moderate antioxidant activity and a low cytotoxic effect in HCT 116 cells. R_EO presented a significant cytotoxicity in colorectal cancer cells and a low antimicrobial effect. The cytotoxic effect on non-cancerous cell line HaCaT was not significant for both essential oils. These results may provide an experimental basis for further research concerning the potential use of M_EO and R_EO for anticancer treatment.

## 1. Introduction

Colorectal cancer is a heterogenous pathology characterized by a landscape of genomic and epigenomic alterations (chromosomal and microsatellite instability, and aberrant methylation) [1,2] and occupies the third place as the most frequently diagnosed type of cancer (10%), and the second place in terms of mortality (9.4%) after lung cancer [3]. The complexity of colorectal cancer stands not only in the molecular heterogeneity (genetic factors), but also in other factors that are considered modifiable as: environmental factors (dietary habits, sedentarism, obesity, etc.), gut microbiota and chronic colonic inflammation [4]. Despite the progress recorded in terms of pathophysiology insights and treatment approaches (radiotherapy, immunotherapy, endoscopic and surgical excision, targeted therapy, palliative chemotherapy, extensive surgery, and local ablative therapies), the incidence and the mortality rates are still increased worldwide [5]. Even though colorectal cancer is known as one of the most preventable tumors (due to its origin from benign neoplasms) [6] once it advances becomes one of the most lethal types of cancer [7]. The paucity of effective chemotherapeutic approaches, the development of resistance to therapy, the lack of specificity towards cancer cells and the side effects derived from this, highlight the need for novel alternatives [8]. Several natural methods were approached as alternative therapies in colorectal cancer among with the assessment of essential oils’ (EOs) anticancer potential and several preclinical studies results seem promising [4,7,9,10,11,12], still this subject is far from being elucidated. 

EOs are defined as secondary metabolites of aromatic plants with a complex composition consisting in a mixture of different chemical compounds (oxygenated compounds and hydrocarbons), that exert key roles in the protection of plants against different assailants (microorganisms, insects, and herbivores), in the signal transduction pathways within the plant, and in human health due to the plurality of innate pharmacological properties [4,8,13]. The plethora of EOs’ biological effects is derived from the mixture of bioactive compounds found in the essential oils’ composition, that are responsible for the initiation of different cellular responses. Among the most studied and described therapeutical effects of EOs, are antimicrobial, antioxidant, antidiabetic, preventive in cardiovascular diseases, and a special interested was granted to the anticancer potential [4,8,13,14]. 

Previous in vitro and in vivo studies reported the anticancer potential of EOs against different types of cancer, as: lung, breast, melanoma, mouth, liver, colon, brain, prostate, pancreatic cancer, and leukemia [8,14]. An advantage of EOs in the fight against cancer could be considered their lipophilic character that facilitates their crossing through cells’ membranes and reaching into cells’ inner side [14]. Two EOs of interest in terms of biological activity and anticancer potential are *Mentha piperita* L. (*M. piperita*) and *Rosmarinus officinalis* L. (*R. officinalis*). 

*Mentha piperita* L. commonly named peppermint is a native genus of the Mediterranean region [15]. The essential oil of this herb is used in fragrances, flavors, and pharmaceuticals [16]. European Medicines Agency (EMA) recognizes multiple pharmacological properties of *Mentha piperita* L. essential oil, including to relief symptoms in coughs and colds, to treat muscle pain and neuralgic pain, and the ones correlated with gastrointestinal disorders (relief of abdominal pain, flatulence, repletion, obstipation, and diarrhea). Other biological effects identified for *M. piperita* are insecticidal, antibacterial, antiviral, antiallergenic, antioxidant, and cytotoxic activities [17,18,19,20]. Peppermint essential oil (M_EO) for inhalation use is effective for relieving symptoms like nausea, vomiting and anorexia form patients receiving chemotherapy [21]. In addition, M_EO exerted an important activity on HeLa cells due to its important protective and bioactive properties [22]. 

*Rosmarinus officinalis* L. is a medicinal herb that belongs to the *Lamiaceae* family and is commonly called rosemary. Rosemary is commonly used is in the culinary field, due to its aromatic properties [23]. Rosemary’s essential oils (R_EO) and extracts obtained from flowers and leaves are used in dermatology to treat minor wounds and rashes, in neurology for headache and circulation problems, in pulmonary field as expectorant and in gastrointestinal disorders as anti-dyspeptic, diuretic and antispasmodic in renal colic [24]. The anticancer effect of rosemary has been confirmed in breast, liver, prostate, lung, and leukemia cancer cells [25]. R_EO exhibited high antioxidant, antiradical, and antimicrobial activities [26]. It presents strong antibacterial and a cytotoxic effect against SK-OV-3 ovarian adenocarcinoma, HO-8910 ovarian carcinoma and Bel-7402 hepatocellular carcinoma [27]. 

Even though the anticancer potential of M_EO and R_EO was proved in different types of cancer (cervical, breast, lung, ovarian, hepatocellular, etc), the role of these oils in colorectal cancer is unexplored. On this basis, the present study was subjected to investigate two commercial essential oils of *M. piperita* and *R. officinalis* (Fares Biovital Laboratories) in terms of chemical composition, antioxidant and antimicrobial properties, and in vitro cytotoxic potential in a human colorectal cancer cell line—HCT 116. 

## 2. Results

### 2.1. GC-MS Analysis and Antioxidant Potential

The chemical composition of *Mentha piperita* L. essential oil is presented in Table 1. A number of 27 compounds were identified in total, and the main compounds were: menthone (28.970%), menthol (22.396%), eucalyptol (12.556%), mentholacetate (8.436%), mentha-3 ona, cis- (7.044%), limonene (3.816%), caryophyllene (3.760%), piperitone (2.686%), β-pinene (2.204%), α-pinene (1.974%), and menthofuran (1.681%).

In general, the chemical compounds of essential oils could be categorized into monoterpenes (oxygenated and hydrocarbons) and sesquiterpenes (hydrocarbons and oxygenated). The results revealed that chemical composition of the M_EOs is characterized mainly by the presence of monoterpenes (70.36%), followed by a mixture of other compounds, while sesquiterpenes were also present in small quantities (11.11%).

Regarding the chemical composition of *Rosmarinus Officinalis* L. essential oil, 23 compounds were identified as presented in Table 2. The compounds identified in concentrations greater than 1% were: eucalyptol (33.592%), α-pinene (12.239%), L-camphor (12.222%), β-Thujene (9.709%), β-pinene (9.435%), camphene (5.723%), caryophyllene (2.859%), p-menth-1-en-8-ol (2.074%), borneol (1.999%), *p*-cymol (1.743%), β -Myrcene and α -Phellandrene (1.546%), limonene (1.474%), and bornyl acetate (1.186%).

The results revealed that chemical composition of the R_EOs is characterized mainly by the presence of monoterpenes (82.61%) and low levels of sesquiterpenes (13.04%) and small quantities of other compounds (4.34%).

The results related to antioxidant activity are presented in Table 3 as inhibition percentage (IP) (%) calculated with the formula described in literature [29], normalized to control (ascorbic acid—a well-known antioxidant compound). Half maximal effective concentration (EC_50_), characterizing the antioxidant potency as evaluated by the DPPH (2,2-diphenyl-1-picrylhydrazyl) assay, were as follows: M_EO = 45.5 µg/mL and R_EO = 74.06 µg/mL. These data represent the mean values ± SD (standard deviation) of three independent experiments performed in triplicate. One-way ANOVA test was performed to determine the statistical differences in rapport with control group followed by Dunnett’s multiple comparisons post-test (**** *p* < 0.0001).

### 2.2. Antimicrobial Activity

In Table 4 and Table 5 are presented the results obtained after the assessment of antimicrobial activity. The data showed that M_EO, used in concentration of 10 μg/mL (M_EO_1), 5 μg/mL (M_EO_2), and 2.5 μg/mL (M_EO_3) exerted significant antimicrobial activity compared to R_EO (tested at the same concentrations), especially in Gram-positive bacteria. The most notable antibacterial effect exerted by M_EO was against Gram positive bacteria *Streptococcus pyogenes* [inhibition zone (IZ) = 33.33 mm, MIC (minimum inhibitory concentration) and MBC (minimum bactericidal concentration) 1.25 µg/mL, at 10 μg/mL)] and the weakest against Gram negative bacteria *Pseudomonas aeruginosa* (11.33 mm, at 10 μg/mL) while the antifungal effect was significant on both fungi *Candida albicans* [IZ = 32.33 mm, MIC and MFC (minimum fungicidal concentration) 1.25 µg/mL at 10 μg/mL] and *Candida parapsilosis* (IZ = 31.33 mm, MIC and MFC 1.25 µg/mL at 10 μg/mL). R_EO, used in concentration of 10 μg/mL, exhibited a more pronounced effect than the positive control against *Pseudomonas aeruginosa* (IZ = 20.33 mm, MIC, and MBC 5 µg/mL at 10 μg/mL). In almost all cases, MBC was equal to MIC, with the highest value for R_EO, at the highest concentration (10 μg/mL), against *P. aeruginosa*. The exceptions were observed in the case of R_EO_1 against *S. pyogenes* where MBC was ten time higher than MIC and in the case of M_EO_3 against *C. albicans* where MFC was double than MIC (Table 5).

### 2.3. The Cytotoxic Effect of Mentha piperita L. Essential Oil 

**Figure 1:**
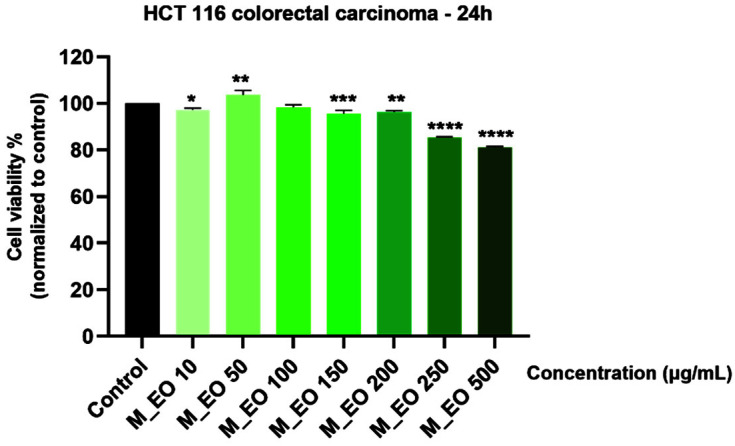
Viability assessment of Mentha piperita L. essential oil—M_EO (10, 50, 100, 150, 200, 250 and 500 μg/mL) in HCT 116 cells at 24 h post-stimulation by MTT assay. The results are presented as cell viability percentage (%) normalized to control (non-stimulated) cells. These data represent the mean values ± SD of three independent experiments performed in triplicate. One-way ANOVA test was performed to determine the statistical differences in rapport with control group followed by Dunnett’s multiple comparisons post-test (* *p* < 0.05, ** *p* < 0.01, *** *p* < 0.001 and **** *p* < 0.0001).

In order to evaluate the potential cytotoxic effects of M_EO on HCT 116 cells, the cells were incubated with different concentrations (10, 50, 100, 150, 200, 250 and 500 μg/mL) for 24 h and the cell viability percentage was calculated using 3-(4,5-dimethylthiazol-2-yl)-2,5-diphenyltetrazolium bromide (MTT) assay. It was noticed a decrease in the cell viability percentages starting with the concentration of 150 μg/mL, and the highest decrease was calculated for the highest concentration tested—500 μg/mL (81.15%—Figure 1). These results indicate a mild/low cytotoxicity induced by M_EO in colorectal cancer cells.

**Figure 2:**
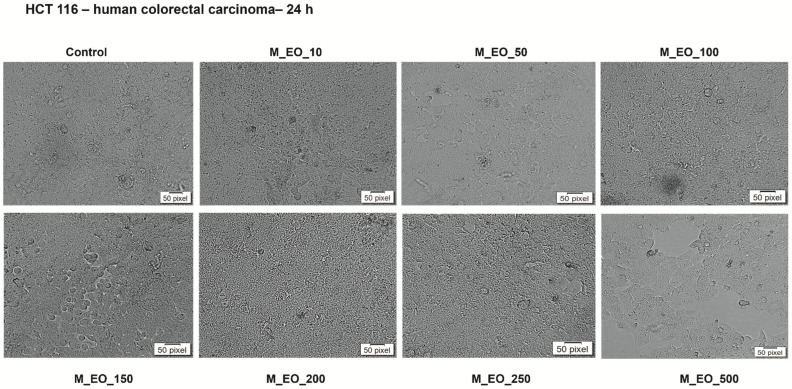
Morphological aspect of HCT 116 cells after treatment for 24 h with *Mentha piperita* L. essential oil—M_EO (10, 50, 100, 150, 200, 250 and 500 μg/mL).

Another aspect investigated in the present study was the impact of M_EO on HCT 116 cells’ morphology (Figure 2), an important marker in establishing the type of induced cytotoxity. The pictures taken at 24 h post-treatment indicated the lack of changes in cells shape, number and confluency at low concentrations (10–100 μg/mL), their aspect being similar to the one of control cells (unstimulated cells), whereas with increasing concentrations (150–500 μg/mL) were detected several round and floating cells and at the highest concentration—500 μg/mL, the confluency was also reduced as compared to control cells (Figure 2).

**Figure 3:**
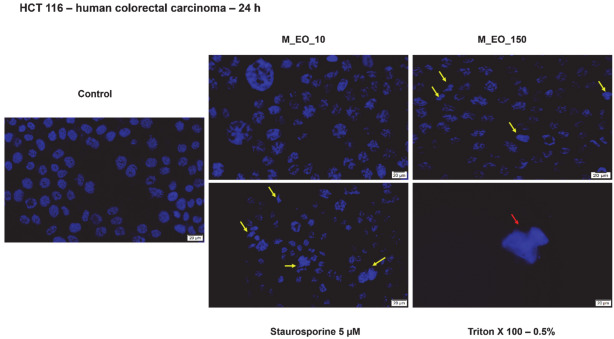
Colorectal cancer cells—HCT 116 nuclei stained with Hoechst 33342 dye after a 24 h treatment with Mentha piperita L. essential oil—M_EO (10 and 150 μg/mL). Staurosporine (5 µM) was used as the positive control for apoptotic changes at nuclear level and Triton X-100 (0.5%) for necrosis. The yellow arrows indicate signs of apoptosis, and the red arrow shows signs of necrosis. The scale bar was 20 µm.

Based on data from cell viability test and cells’ morphology evaluation, it was verified if the cytotoxic signs induced by M_EO treatment were specific for apoptosis or necrosis by the means of Hoechst staining (Figure 3). The concentrations selected for this experiment were the lowest—10 μg/mL and the concentration responsible for the first signs of cytotoxicity—150 μg/mL. The nuclei of the HCT 116 cells were not affected by the lowest concentration of M_EO presenting a round shape similar to control cells, still the 150 μg/mL concentration triggered some apoptotic specific signs (nuclear shrinking and fragmentation—yellow arrows) as the ones described for Staurosporine solution (control positive for apoptosis). No signs of necrosis were detected in the groups of cells incubated with M_EO, as seen in cells exposed to Triton X-100 solution (control positive for necrosis) (Figure 3).

**Figure 4:**
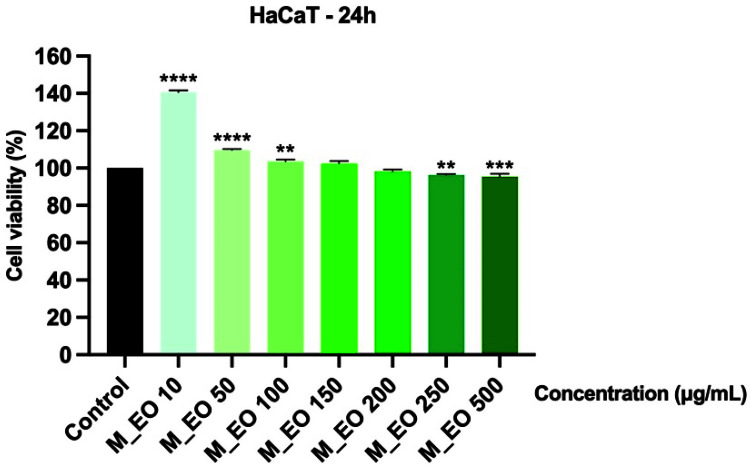
Viability assessment of Mentha piperita L. essential oil—M_EO (10, 50, 100, 150, 200, 250 and 500 μg/mL) in HaCaT cells at 24 h post-stimulation by MTT assay. The results are presented as cell viability percentage (%) normalized to control (non-stimulated) cells. These data represent the mean values ± SD of three independent experiments performed in triplicate. One-way ANOVA test was performed to determine the statistical differences in rapport with control group followed by Dunnett’s multiple comparisons post-test (** *p* < 0.01, *** *p* < 0.001 and **** *p* < 0.0001).

In order to verify the selective cytotoxicity of M_EO on cancer cells, human immortalized keratinocytes—HaCaT were incubated for 24 h with M_EO (10, 50, 100, 150, 200, 250 and 500 μg/mL) and analyzed in terms of cell viability and celullar morphology. The viability assessment indicated that the concentrations tested had no toxic impact on the cells’ viability percentage, at the highest concentration—500 μg/mL being calculated a viability percentage higher than 95% (Figure 4). 

**Figure 5:**
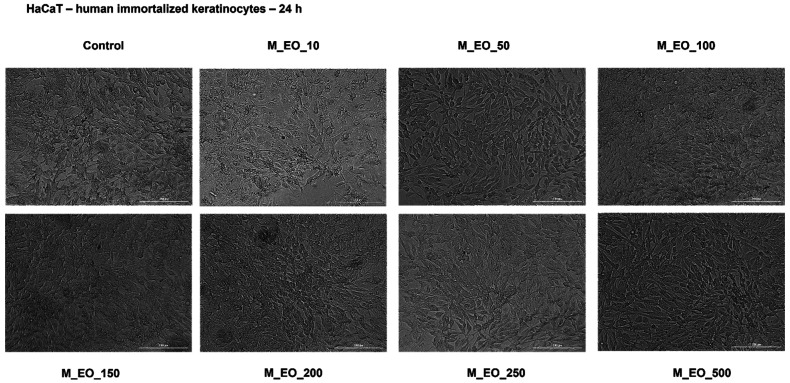
Morphological aspect of HaCaT cells after treatment for 24 h with Mentha piperita L. essential oil—M_EO (10, 50, 100, 150, 200, 250 and 500 μg/mL). The scale bar was 200 μm.

As regards the effect on HaCaT cells’ morphology, as it can be seen in Figure 5, M_EO treatment did not affect cells’ shape, cell-cell adherence and confluency, data that support the cell viability results. Based on these data, it can be stated that M_EO had no toxic effects on HaCaT cells.

### 2.4. The Cytotoxic Effects of Rosmarinus officinalis L. Essential Oil

**Figure 6:**
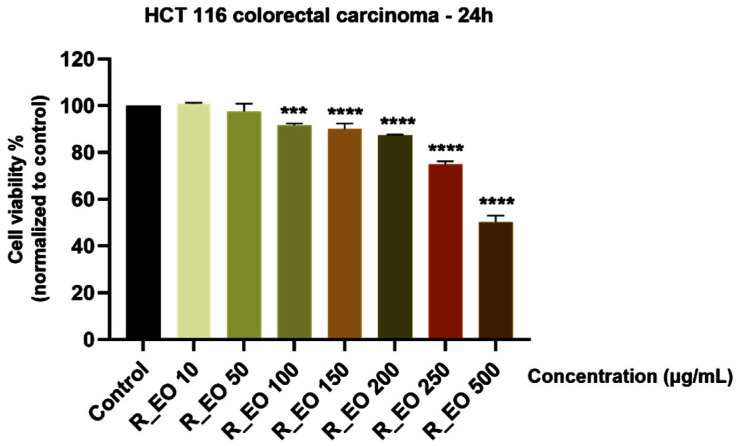
Viability assessment of Rosmarinus officinalis L. essential oil—R_EO (10, 50, 100, 150, 200, 250 and 500 μg/mL) in HCT 116 cells at 24 h post-stimulation by MTT assay. The results are presented as cell viability percentage (%) normalized to control (non-stimulated) cells. These data represent the mean values ± SD of three independent experiments performed in triplicate. One-way ANOVA test was performed to determine the statistical differences in rapport with control group followed by Dunnett’s multiple comparisons post-test (*** *p* < 0.001 and **** *p* < 0.0001).

A similar experimental protocol was applied for the evaluation of R_EO cytotoxic potential in HCT 116 cells as the one described above for M_EO, and it can be observed that R_EO induced a stronger reduction of cell viability percentage in cancer cells as compared to M_EO (Figure 6). R_EO induced a dose-dependent decrease of cell viability percentage, the lowest percentage of viable cells (50.25%) being calculated for the highest concentration tested—500 μg/mL.

**Figure 7:**
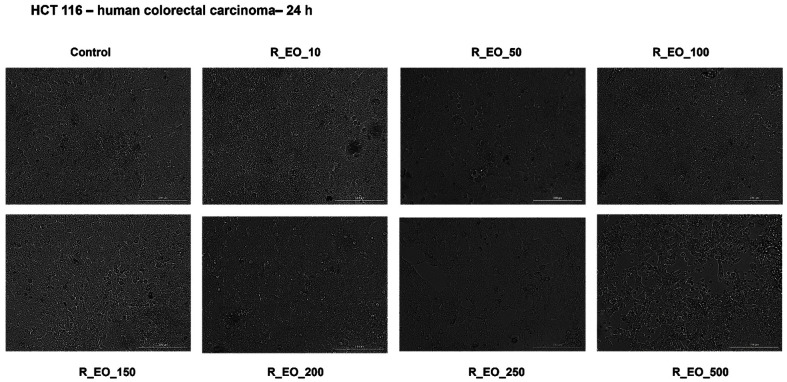
Morphological aspect of HCT 116 cells after treatment for 24 h with Rosmarinus officinalis L. essential oil—R_EO (10, 50, 100, 150, 200, 250 and 500 μg/mL). The scale bar was 200 μm.

Since R_EO treatment triggered a dose-dependent reduction of HCT 116 cells’ viability, we also analyzed its impact on cells’ morphology (Figure 7). No signs of roundish or detached cells and affected adherence were noticed in the group of cells incubated with low concentrations of R_EO (10–100 μg/mL), their aspect being comparable with the control cells. Several roundish and detached cells, but unmodified adherence was noticed at 150 and 200 μg/mL, but the highest concentrations—250 and 500 μg/mL induced significant morphological changes as round cells floating, loss of cell–cell adhesions, loss of adherence, reduced confluence, and cellular debris (Figure 7), specific signs of cytotoxicity.

**Figure 8:**
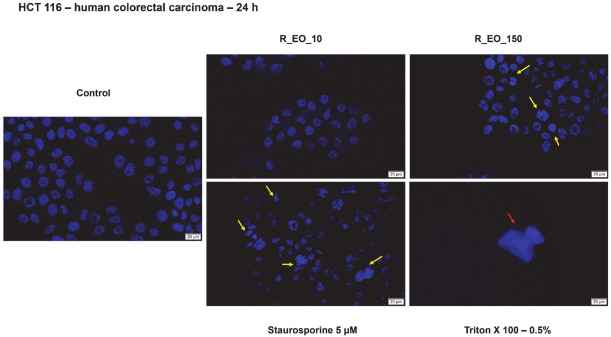
Colorectal cancer cells—HCT 116 nuclei stained with Hoechst 33342 dye after a 24 h treatment with Rosmarinus officinalis L. essential oil—R_EO (10 and 150 μg/mL). Staurosporine (5 µM) was used as the positive control for apoptotic changes at nuclear level and Triton X-100 (0.5%) for necrosis. The yellow arrows indicate signs of apoptosis, and the red arrow shows signs of necrosis. The scale bar was 20 µm.

For the Hoechst 33,342 staining assay of R_EO were selected the same concentrations (10 and 150 μg/mL) as for M_EO, the selection being done on the same consideration as in the case of M_EO. The lowest concentration of R_EO—10 μg/mL had no impact on HCT 116 cells nuclei, whereas the highest concentration verified—150 μg/mL was associated with the presence of apoptotic cells characterized by the presence of nuclear shrinkage, nuclear fragmentation, chromatin condensation (Figure 8, yellow arrows). No signs of necrosis were detected in the cells treated with R_EO.

**Figure 9:**
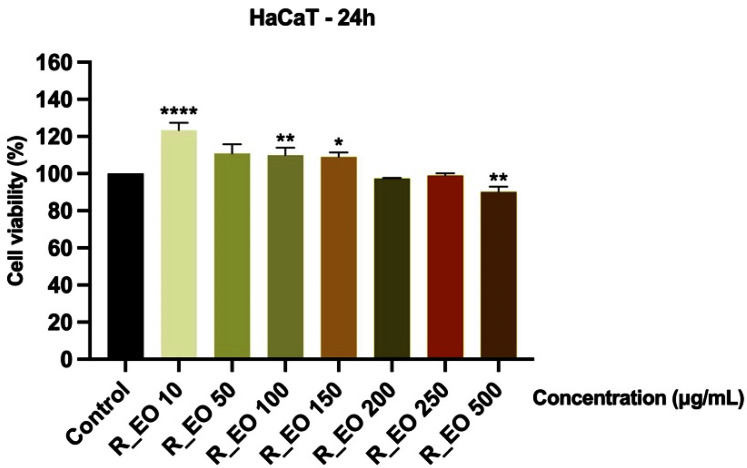
Viability assessment of Rosmarinus officinalis L. essential oil—R_EO (10, 50, 100, 150, 200, 250 and 500 μg/mL) in HaCaT cells at 24 h post-stimulation by MTT assay. The results are presented as cell viability percentage (%) normalized to control (non-stimulated) cells. These data represent the mean values ± SD of three independent experiments performed in triplicate. One-way ANOVA test was performed to determine the statistical differences in rapport with control group followed by Dunnett’s multiple comparisons post-test (* *p* < 0.05, ** *p* < 0.01 and **** *p* < 0.0001).

**Figure 10:**
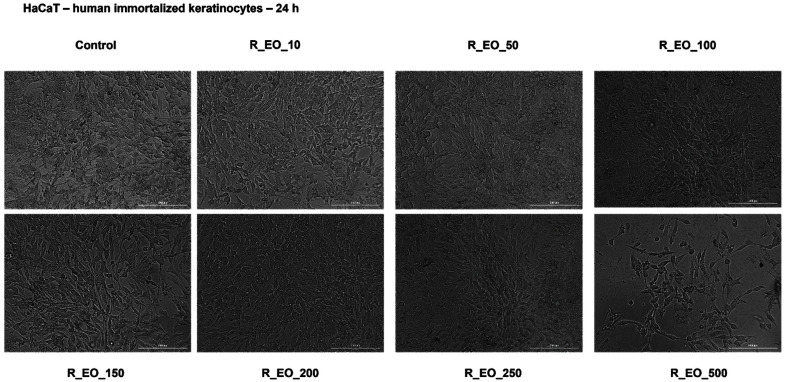
Morphological aspect of HaCaT cells after treatment for 24 h with Rosmarinus officinalis L. essential oil—R_EO (10, 50, 100, 150, 200, 250 and 500 μg/mL). The scale bar was 200 μm.

The in vitro potential cytotoxicity of R_EO was also tested using the HaCaT non-cancerous cell line. After a 24 h incubation with the different concentrations of R_EO (10, 50, 100, 150, 200, 250 and 500 μg/mL), it was observed a slight decrease of cells’ viability (around 90%) and changes in cells’ confluency only at the highest concentration—500 μg/mL (Figure 9 and Figure 10). 

The shape of the HaCaT cells was not modified following the R_EO treatment and no signs of cytotoxicity (roundish cells floating and cell debris) were detected (Figure 10).

## 3. Discussion

Colorectal cancer represents one of the leading causes of death worldwide and in the last years, the interest of researchers was oriented towards the potential role of EOs in the prevention and treatment of this type of cancer and other colonic-related pathologies [4]. The beneficial effects of EOs in colonic pathophysiology described were an anti-inflammatory activity by modulating different signaling pathways involved in acute and chronic inflammation, the antioxidant potential, the interference with the intestinal microbiota, chronic gut inflammation and intestinal microbiota representing important environmental factors in the development of colorectal cancer, and the cytotoxic effect [4].

Based on the different bioactive constituents, EOs act as multi-target molecules becoming potential alternatives for chemoprevention and cancer complementary treatment [30]. 

The anticancer potential of *Mentha piperita* L. and *Rosmarinus officinalis* L. essential oils was previously proved against different cancer types, as: human cervix carcinoma (HeLa cells), human lung carcinoma (A549 and SPC-A1 cells), human non-small cell lung cancer (H1299 cells), human breast adenocarcinoma (MCF-7 cells) [8,23,31,32,33,34], human leukemia (K562 cells) [32], human gastric cancer (SGC-7901 cells) [32], oral squamous carcinoma (SCC-25 cells) [35], human hepatoma (HepG2 and Bel-7402 cells) [27,36], and ovarian cancer (A2780, K-OV-3 and HO-8910 cells) [37,38], still the data regarding the impact of these oils in colorectal cancer are rather scarce. 

Therefore, we analyzed the effects of two EOs commercially available (M_EO and R_EO) in colorectal cancer cells—HCT 116 in terms of chemical composition, antioxidant potential, antimicrobial activity, and cytotoxicity.

The results obtained in the present study from the GC-MS analysis of the chemical composition are in accordance with those presented in the literature. The major compounds of M_EO were menthone and menthol (representing around 51% of total compounds, see Table 1), identified in other studies in significant quantities, in various samples from different geographical locations [39,40,41]. In the case of R_EO the major compounds were eucalyptol, α-pinene and camphor (representing around 58% of total compounds, see Table 2). As in the case of M_EO, the composition of R_EO differs significantly from one area to another, in some place’s eucalyptol being identified as the main compound (as in this case), in others being identified in very small quantities [42]. It is well known that the chemical composition of essential oils is influenced, especially from a quantitative point of view, by several factors such as: plant species, variety, age, part collected, origin, climate, soil, agrochemicals used, processing methods etc. Phytochemical characterization is of major importance both in order to establish the variety of components that fluctuates from one sample to another and for the reproducibility and accuracy of data [42].

Biological activity is closely related to chemical composition, and the existence of certain functional groups significantly influences this activity [43]. M_EO samples expressed varied antimicrobial activity against the microbial strains tested, the values being in some cases significantly higher than the positive control (e.g., *S. mutans*, *S. pyogenes* and *Candida*) and also compared to R_EO which did not show significant activity in these strains. The greatest inhibition induced by M_EO was observed against *S. pyogenes*, confirming the data from previous studies which mention the higher susceptibility of Gram-positive species to EOs [44]. 

According to the latest EMA’s assessment report on *Mentha x piperita* L. aetheroleum, the peppermint essential oil’s medicinal use related to gastrointestinal illnesses is as symptomatic treatment for mild spasm of the gastrointestinal and bile tract, flatulence, and irritable bowel syndrome (abdominal pain, obstipation, diarrhea, and repletion) [45]. Moreover, several studies support the use of peppermint EO for its antiemetic effect in chemotherapy-induced nausea and vomiting [21,46]. In recent years, there has been a significant increase in the consumption of ultra-processed foods at the expense of natural foods, a fact that decisively influences the state of adequate immunity. In some in vivo studies, the beneficial effect of essential oils on the microbial composition of the large intestine has been demonstrated by increasing the number of probiotic bacteria [47]. Several studies have focused on the synergistic antimicrobial effects exerted by different classes of compounds while regarding the association of essential oils (recognized antimicrobial) and probiotics (recognized for their ability to delay the growth of microorganisms), the results are promising [48].

To the best of our knowledge, the data regarding the cytotoxic effect of M_EO in colon cancer cells are lacking. In the view of these facts, the present study proposed the analysis of the M_EO potential cytotoxicity in HCT 116 colon cancer cells by assessing its impact on cells’ viability, cellular and nuclear morphology. 

Our results indicate that M_EO (10–500 µg/mL) treatment for 24 h exhibited a low cytotoxicity in HCT 116 cells characterized by a dose-dependent reduction of cells’ viability percentage (Figure 1), significant changes in cells’ morphology (round shape, cells’ floating and reduced confluence) only at the highest concentration tested—500 µg/mL (Figure 2), and apoptotic-like signs starting from 150 µg/mL (Figure 3). As stated in previous studies, the potency of the cytotoxic effect of M_EO may vary dependent on the type of cancer cell lines tested and EOs composition, as: (i) highly cytotoxic—human lung carcinoma SPC-A1, human gastric cancer SGC-7901, and human leukemia K562 (IC_50_ = 10.89 µg/mL; 16.16 µg/mL and 38.76 µg/mL, respectively) cells [32], (ii) moderate cytotoxic—human cervical (HeLa—IC_50_ = 165.24 ± 4.40 c/w) and lung (A549—IC_50_ = 183.00 ± 23.10 c/w) cancer cells [31], rat glioma (C6 cells) [49], and (iii) inactive—hepatocellular carcinoma BEL-7402 cells [32]. Different studies have analyzed the effects of monoterpenes on colon tumor cells [4]. Following the stimulation of MRC-5, HT-29 and HCT 116 cells with camphor, eucalyptol, thujone, it was observed that the most sensitive cells to the action of the compounds are the HCT 116 cells (the IC_50_ values determined for these compounds being 4.5, 4 and 1 mM) [50].

Another aspect verified was the potential toxicity of M_EO on a non-cancerous cell line—HaCaT (human immortalized keratinocytes). Even at the highest concentration tested—500 µg/mL, no significant changes were noticed in HaCaT cells’ shape and confluence, and the cells’ viability did not decrease under the percentage of 95% (Figure 4 and Figure 5). The very low toxicity of M_EO in non-cancerous cells was also stated in other studies [31,32,49]. Moreover, based on the EMA’s report, the oral lethal dose (LD_50_) of peppermint oil analyzed in fasted Wistar male rats was found to be 4441 ± 653 mg/kg after 24 h and 2426 mg/kg after 48 h [45], and the No Observed Adverse Effect Level (NOAEL) for oral administration of menthol (the main component of the peppermint commercial essential oil tested) was established at 188 mg/kg bw/day [4]. Several side-effects of peppermint essential oil observed in clinical trials were presented in EMA’s report, as follows: allergic reactions to menthol, headaches, bradycardia, muscle tremor, ataxia, and anaphylactic shock (after oral use), skin rash, contact dermatitis and eye irritation (after cutaneous use), and broncho- and laryngoconstriction (after inhalation). To reduce the rate of appearance of this kind of effects were established the categories of persons that have contraindications to the use of peppermint essential oil that comprise: patients with liver disease, cholangitis, achlorhydria, gallstones and any other biliary disorders and children under 2 years of age [45].

The other essential oil tested in the present study was the *Rosmarinus officinalis* L. essential oil (R_EO). Multiple biological properties of *Rosmarinus officinalis* L. essential oil were shown in previous studies, including antioxidant and antimicrobial effects (being used as biopreservative in food industry), anti-inflammatory, antidepressant, antinociceptive, DNA-protective, anticancer, and based on the EMA’s report R_EO is recommended for the treatment of dyspepsia and mild spasmodic pathologies of the gastrointestinal tract [27,51,52]. As in the case of M_EO, the reports about R_EO cytotoxicity in colorectal cancer are scanty.

In terms of cytotoxicity, our results indicated that R_EO proved to be more active on HCT 116 colon cancer cells as compared to M_EO, the cells’ viability percentage at the highest concentration tested—500 µg/mL being around 50% (Figure 6). The decrease of HCT 116 cells’ viability was associated with significant changes in cells shape, cell-cell adhesion, and confluency (starting at the concentration of 150 μg/mL and becoming more evident with increasing concentrations—Figure 7). The signs of cytotoxicity observed in the morphology studies were confirmed by the nuclear staining assay that highlighted the presence of apoptotic-like features, as nuclear shrinking and fragmentation, and chromatin condensation, features that were not detected in control cells or in cells treated with the lowest concentration—10 µg/mL (Figure 8). Based on the data from the literature, R_EO showed cytotoxicity against different cancer cell lines, such as: human lung carcinoma (A549, IC_50_ = 3.06 µg/mL), human breast cancer (MCF-7, IC_50_ = 7.38 µg/mL) [8,33,53], human hepatoma (HepG2, DNA fragmentation and cell cycle arrest) [43], human cervical cancer and breast cancer (HeLa, IC_50_ = 0.011 µL/mL; MCF-7, IC_50_ = 0.253 µL/mL, inhibition of proliferation and migration) [23], and ovarian cancer (A2780, K-OV-3 and HO-8910 cell lines, reduction of cell growth) [27,36]. Studies conducted both in vitro and in vivo have shown that eucalyptol suppressed the proliferation of human colorectal cancer by inducing apoptosis [54].

As regards the impact of R_EO on HaCaT non-cancerous cells, it was recorded a slight decrease in cells’ viability only at the 500 µg/mL concentration (around 90%) and a reduction of cells’ confluency (Figure 9 and Figure 10). These results are in agreement with the data found in the literature regarding the lack/very low toxicity of R_EO in healthy cells as compared to tumor cells, including hepatocytes (hepatoprotective effects against CCl_4_-induced toxicity) [52], human peripheral lymphocytes [36] and HUVEC cells [40]. Even though the rosemary preparations (including essential oil) are considered safe and devoid of toxicity in recommended doses, in EMA’s report regarding rosemary oil are stated some precautions as regards the use of this oil during pregnancy and lactation (lack of data), irritant potential (after cutaneous use) and is contraindicated to patients diagnosed with duct obstruction, liver diseases, gall stones, cholangitis and other biliary disorders. The compounds found in the composition of rosemary oil associated with severe side-effects are camphor (epileptic seizures in adults and death in children) and carnosol (contact dermatitis and occupational asthma) [52].

Different studies have shown the complementary effects obtained with the association between essential oils and 5-fluorouracil (5-FU). In mice transplanted with sarcoma 180 tumor cells and treated with peppermint essential oil/5-FU (50 and 100 mg/kg/day respectively 10 mg/kg/day) showed a greater inhibition of tumor growth and a less severe leukopenia compared to treatment with only 5-FU [55]. The rosemary extract presents dose-dependent anticarcinogenic activities and exerts a synergistic effect in combination with 5-FU on colon cancer cells [56]. Other studies have highlighted that the main mode of action of cyclic terpenes (e.g., α-pinene, carvone, eucalyptol) is based on their interaction with the lipids of the intercellular stratum corneum (SC) thus increasing the diffusivity of 5-fluorouracil through human epidermal membranes [57]. One of the major components in peppermint essential oil, menthol, has been shown to be at least as active as 5-FU in melanoma tumor cells [58]. Another approach involved combining in vitro permeation studies and coarse-grained molecular dynamics to investigate the effects of menthol and borneol on 5-FU activity. The results showed that both substances exerted penetration-enhancing effects on 5-FU, menthol involving disruption of the stratum corneum bilayer, and borneol involving multiple mechanisms, including disruption of the SC bilayer, increasing the diffusion coefficient of 5-FU and inducing the formation of transient pores [59]. The studies conducted to evaluate the blocking of differentiation of Caco-2 cells with the aim of sensitization to treatment with 5-fluorouracil showed the synergistic effect obtained when combining geraniol with 5-FU. Cytotoxicity induced by the chemotherapeutic agent was enhanced in the presence of geraniol, the effect resulting from the facilitated transport of 5-FU and the blocking of morphological and functional differentiation of cancer cells [60]. In another animal model study (TC-118 human tumors transplanted in Swiss nu/nu mice) it was shown that the combined administration of 5-fluorouracil (20 mg/kg) and geraniol (150 mg/kg) produced a reduction by more than 50% of the tumor volume, compared to a reduction of approximately 25% in the treatment with geraniol and the lack of tumor reduction in the treatment with only 5-FU [61]. These data underline the fact that essential oils can improve the therapeutic effectiveness of chemotherapy drugs. 

## 4. Materials and Methods

### 4.1. Reagents

The essential oils R_EO and M_EO analyzed in the present study were purchased from Fares Biovital Laboratories (Hunedoara, Romania) and present the following identification items: M_EO—Ulei Menta R 20, 10 mL for internal use (batch number: 36/19, reference number: 5197/21.01.2011), and R_EO—Ulei Rozmarin A11, 10 mL for internal use batch number: 10/19, reference number: 6040/14.10.2011).

The reagents applied to perform the experimental part were: N-hexane (Merck, Darmstadt, Germany), 2,2-diphenyl-1-picrylhydrazyl (DPPH, Sigma Aldrich, Merck KgaA, Darmstadt, Germany), ascorbic acid (LachNer Company, Prague, Czech Republic), methanol 99% (Chimopar, Bucuresti, Romania), Columbia blood agar, Sabouraud agar and Mueller-Hinton agar (BioMerieux, Marcy-l’Étoil, France), gentamycin (GM) and fluconazole (FCZ) disks (BioRad, Marnes-la-Coquette, France), phosphate saline buffer (PBS), fetal bovine serum (FBS), penicillin/streptomycin, trypsin-EDTA solution, dimethyl sulfoxide (DMSO) and 3-(4,5-dimethylthiazol-2-yl)-2,5-diphenyltetrazolium bromide (MTT) reagent (Sigma Aldrich, Merck KgaA, Darmstadt, Germany), and Hoechst 33342 nuclear staining assay (Invitrogen, Carlsbad, CA, USA). The cell culture media, McCoy’s 5A Medium (ATCC^®^ 30-2007™) and Dulbecco’s modified Eagle Medium (DMEM) high glucose—4.5 g/L (ATCC^®^ 30-2002™) were purchased from ATCC (American Type Cell Collection, Lomianki, Poland). All reagents complied with the analytical standard purity and were used according to the manufacturers’ recommendations.

### 4.2. Gas Chromatography-Mass Spectrometry (GC-MS) Analysis

The chemical composition of the EOs was identified by GC-MS analysis. GS/MS QP 2010 Plus (Shimadzu, Kyoto, Japan) connected at AT WAX 30 m × 0.32 mm × 1 μm capillary column was utilized and helium with a flow rate of 1 mL/min was the carrier gas. Briefly, the following steps have been applied: (i) temperature program—initially 40 °C followed by an increase of 5 °C/min until reaching the final temperature of 210 °C (for 5 min), injector temperature 250 °C, ion source 220 °C; (ii) injection volume 1 μL at a split ratio of 1:50; dilution of samples 1:10 (*v*/*v*), solvent n-hexane. The NIST 02 and Wiley 275 libraries spectra databases were employed to compare and identify the volatile compounds. The linear retention indices (LRI) were determined according to Van den Dool and Kratz formula, related to a homologous series of n-alkanes (C8–C24) [62].

### 4.3. Assessment of the Antioxidant Capacity

The antioxidant capacity of the EOs was evaluated using the DPPH assay, according to the method described in the literature [29,51] with small modifications: (i) preparation of DPPH 0.1 mM solution (in methanol); (ii) sample dilution (in methanol) to obtain different concentrations (5, 10, 25, 50, 75, 100 and 200 µg/mL); (iii) absorbance reading at 517 nm (for 20 min) using an UviLine 9400 spectrophotometer from SI Analytics (Mainz, Germany). The positive control was represented by a methanol solution of ascorbic acid 0.4 mg/mL. The inhibition percentage of DPPH free radical, expressed as (IP%), was calculated with the formula presented in the literature [29].

### 4.4. Antimicrobial Activity

The EOs antibacterial activity was tested against *Streptococcus mutans* (ATCC 35668™), *Streptococcus pyogenes* (ATCC 19615™), *Staphylococcus aureus* (ATCC 25923™), *Escherichia coli* (ATCC 25922™), *Pseudomonas aeruginosa* (ATCC 27853™) and for antifungal activity against *Candida albicans* (ATCC 90029™) and *Candida parapsilosis* (ATCC 22019™) (all strains were acquired from American Type Culture Collection, Microbiologics, Molsheim, France) using the Disk diffusion method for susceptibility testing, according to the Standard Rules for Antimicrobial Susceptibility Testing using Impregnated Disks. In brief, the following steps were realized: (i) the Mueller-Hinton agar, supplemented for streptococci with horse blood and ß-NAD, was inoculated with microbial standardized suspension (0.5 Mc Farland); (ii) 10 µL from each sample was added to a blank disk (BioMaxima, Lublin, Poland), placed on top of the agar; (iii) the plates were incubated at 37 °C for 24 h; (iv) the inhibition zone diameters were determined in millimeters. For all bacterial strains were performed triplicate disk-diffusion tests. The positive controls were gentamycin (GM, BioRad, Marnes-la-Coquette, France) for bacteria and fluconazole (FCZ, BioRad, France) for fungi, while negative control was the solvent (ethanol).

The dilution tests were conducted according to previous studies based on the recommendations of the Clinical Laboratory and Standard Institute (CLSI) and the European Committee on Antimicrobial Susceptibility Testing (EUCAST) [63,64,65,66]. The working protocol involved the following steps: (i) obtaining of a serial two-fold dilutions of the compounds, in Mueller-Hinton broth with the microbial inoculum (finally approximate 500,000 microorganisms/mL broth); (ii) after the incubation period of the tubes (at 37 °C for 24 h), the lowest concentration without visible growth (MIC) was determined. The MBC or MFC was considered the lowest concentration which killed 99.9% of the microorganisms and was determined by cultivation of 1 µL suspension, from test tubes with no growth, on Columbia blood agar or Sabouraud agar (BioMerieux, Marcy-l’Étoil, France).

### 4.5. Cell Culture

Two cell lines acquired from ATCC (American Type Cell Collection) and CLS Cell Lines Service GmbH (Eppelheim, Germany), respectively, were used as in vitro models, namely: human colorectal carcinoma cells—HCT 116 (ATCC^®^ CCL-247TM) and immortalized human keratinocytes—HaCaT. The HCT 116 cells were cultured in their specific culture medium—McCoy’s 5A medium, whereas the HaCaT cells [67] required DMEM high glucose—4.5 g/L medium. To obtain the complete medium for culture 10% FBS and 1% antibiotic mixture (100 U/mL penicillin/100 µg/mL streptomycin) were added. The growth of cells was performed in standard conditions: 5% CO_2_ and a temperature of 37 °C in a humidified incubator. 

### 4.6. Cell Viability Evaluation

The impact of EOs on cell viability was assessed using the MTT assay. The experimental protocol was applied according to our previous studies [68] and adapted to the present experimental requirements. The protocol comprised the following steps: (1) HaCaT and HCT 116 cells were cultured in 96-well plates (1 × 10^4^ cells/200 µL complete medium/well) and when the confluence was reached were incubated with different increasing concentrations of EOs (10–500 µg/mL) for a 24 h period; (2) after the 24 h incubation period, the old medium was replaced by fresh culture medium (100 µL/well) and a volume of 10 µL/well of MTT solution (5 mg/mL) was added in each well followed by a 3 h incubation; (3) the formazan crystals formed were dissolved in 100 µL of solubilization buffer provided by the manufacturer during a 30 min repose at room temperature and dark; (4) the reduced MTT was spectrophotometrically analyzed at 570 and 630 nm, using the Cytation 5 (BioTek Instruments Inc., Winooski, VT, USA) microplate reader. All experiments were performed in triplicate. The results were expressed as percentage of viable cells (%). The stock solutions of M_EO and R_EO were prepared in DMSO, and the following dilutions were performed in culture medium.

### 4.7. Assessment of Cellular Morphology

To identify the potential changes/alterations of cellular morphology following EOs treatment (all concentrations tested for viability), the cells were observed using the Olympus IX73 inverted microscope (Olympus, Tokyo, Japan) under bright field and photographed at the end of the 24 h incubation period. The images obtained were analyzed using the cellSens Dimensions v.1.8. Software (Olympus, Tokyo, Japan).

### 4.8. Nuclear Staining Using Hoechst 33342 Dye

To define the type of cytotoxic effect induced by essential oils treatment to colorectal cancer cells—HCT 116 it was used the Hoechst 33342 nuclear staining assay. The implemented protocol was detailed in our previous study [68] and was applied as following: (1) the HCT 116 cells (1 × 10^5^ cells/1.5 mL complete medium/well) were cultured in 12-well plates and incubated for 24 h with two concentrations (10 and 150 µg/mL) of each essential oil: M_EO and R_EO; (2) after 24 h, the old medium was removed, the staining solution 0.5 mL/well (1:2000 in PBS) was added and the cells were incubated for 10 min at room temperature and dark; (3) the cells were washed with PBS (3x) and photographed using the Olympus IX73 inverted microscope (Olympus, Tokyo, Japan). The nuclear staining images were analyzed using the cellSens Dimensions v.1.8. Software (Olympus, Tokyo, Japan). As positive control for apoptosis was used a Staurosporine solution—5 µM (3 h at 37 °C) and for necrosis–Triton X-100 (30 min at 37 °C)—0.5%.

### 4.9. Statistical Analysis

The software used for the statistical analysis was GraphPad Prism version 9.3.1 for Windows (GraphPad Software, San Diego, CA, USA, www.graphpad.com, accessed on 10 May 2022). The results were expressed as means ± SD. The differences between data were compared by performing the one-way ANOVA test and Dunnett’s multiple comparisons post-test. The statistically significant differences between data were labeled with * (* *p* < 0.1; ** *p* < 0.01; *** *p* < 0.001; **** *p* < 0.0001). The normal distribution was also verified by applying the Kolmogorov-Smirnov test (GraphPad Prism version 9.3.1) and the *p* value obtained was *p* < 0.0001.

## 5. Conclusions

Essential oils composed of complex mixtures of hydrophobic and volatile compounds present numerous pharmacological properties but the access to clinical studies is difficult based on their complexity but also on the significant variations between the compositions of the same oil having different geographical origins. Therefore, to obtain complete, reproducible and clinical results, an adequate physico-chemical and biological characterization is mandatory. The present study provides evidence that the *Mentha piperita* L. commercial essential oil (M_EO—rich in monoterpenes −70.36% and sesquiterpenes—11.11% having as major constituents menthone and menthol) exerted potent antimicrobial activity on *S. pyogenes*, poor cytotoxicity in colorectal cancer cells—HCT 166 and no toxic effects in non-cancerous cells—HaCaT. As regards the *Rosmarinus officinalis* L. commercial essential oil (R_EO—rich in monoterpenes −82.61% and sesquiterpenes—13.04% having as major constituents—eucalyptol, α-pinene and camphor), showed low anti-microbial effects, and a significant cytotoxic effect in HCT 116 cells (reduced cell viability and apoptotic-like features) as compared to M_EO. Future research directions require the evaluation of biological effects in association with other classes of compounds (chemotherapy, probiotics) to evaluate synergic actions with a beneficial role in colon tumor diseases.

## Figures and Tables

**Table 1:** The main phytochemical constituents of *Mentha piperita* L. essential oil identified by GC-MS analysis.

Compounds Name	Retention Time	LRI Rep [28]	LRI Exp	Conc. (%)
α-pinene (MH)	6.375	1015–1030	1017	1.974
β-pinene (MH)	8.652	1105–1108	1106	2.204
Thujene (MH)	9.006	1022–1027	1023	0.585
Limonene (MH)	11.286	1196–1199	1196	3.816
Eucalyptol (MH)	11.504	1200–1211	1201	12.556
γ-terpinene (MH)	12.634	1243	1247	0.128
Benzene, tert-butyl-	13.253	1215–1249	1232	0.122
Cyclohexanone, 3-methyl-, (R)-	14.779	1376–1381	1372	0.233
Menthone (MO)	18.745	1438–1448	1446	28.970
Menthofuran	19.171	1477–1503	1491	1.681
Mentha-3 ona, cis-	19.440	-	1502	7.044
α-bourbonene (SH)	20.510	1514–1515	1512	0.339
Linalool (MO)	20.819	1550–1552	1553	0.144
Mentholacetate	21.235	-	1633	8.436
Citronellol acetate	21.721	1645–1662	1655	0.035
Germacrene (SH)	21.895	1684–1702	1694	0.327
Caryophyllene (SH)	22.484	1576–1597	1580	3.760
Pulegone (MO)	23.312	1631	1640	0.869
Estragole	23.674	1624–1685	1662	0.129
β-citral	23.992	1663–1696	1681	0.775
α-caryophyllene (SH)	24.220	1636–1670	1648	0.156
α-terpineol	24.882	1674–1692	1681	0.288
Piperitone	25.204	1715–1743	1723	2.686
β-farnesene	25.711	1660–1662	1658	0.196
Cyclopropane, pentyl-	26.803	-	1649	0.087
Menthol	27.736	1618–1637	1620	22.396
Butanoic acid, 3,7-dimethyl-6-octenyl ester	31.129	1765	1749	0.063
Total				99.999
monoterpenes				70.36
sesquiterpenes				11.11
others				18.52
Total				99.99

GC-MS—gas chromatography—mass spectrometry, LRI rep—linear retention index reported in the literature, LRI exp—linear retention index from the current analysis, MH—hydrogenated monoterpenes, MO—oxygenated monoterpenes, SH—hydrogenated sesquiterpenes.

**Table 2:** The main phytochemical constituents of *Rosmarinus Officinalis* L. essential oil identified by GC-MS analysis.

Compounds Name	Retention Time	LRI Rep [28]	LRI Exp	Conc. (%)
α-Pinene (MH)	6.382	1015–1030	1022	12.239
α-Fenchene (MH)	7.270	1048–1060	1051	0.032
Camphene (MH)	7.512	1060–180	1077	5.723
β-Pinene (MH)	8.657	1105–1108	1100	9.435
β-Thujene (MH)	8.657	1100–1133	1108	9.709
Carane MH	-	-	-	N.D.
β-trans Ocimene (MH)	9.838	1232–1250	1240	0.109
β -Myrcene (MH)	10.180	1155–1164	1154	1.546
α-Phellandrene (MH)	10.180	1153–1168	1158	1.546
1,4-Cineole	-	-	-	N.D.
Limonene (MH)	11.307	1189–1205	1192	1.474
Eucalyptol (MH)	11.542	1198–1211	1201	33.592
γ-Terpinene (MH)	12.616	1243	1252	0.891
*p*-Cymol (MH)	13.253	1261–1283	1268	1.743
Terpinolene (MH)	13.685	1266–1274	1271	0.209
D-Fenchone	-	-	-	N.D.
2-Camphanone	-	-	-	N.D.
L-Camphor (MO)	20.042	1532	1544	12.222
β-Linalool (MO)	20.796	1531–1552	1538	0.552
Bornyl acetate (MO)	21.676	1545–1567	1554	1.186
Caryophyllene SH	22.479	1576–1597	1582	2.859
Estragole	23.673	1624–1685	1658	0.151
α-Caryophyllene (SH)	24.231	1636–1670	1642	0.335
α-Terpinyl acetate (MO)	24.231	1676–1692	1680	0.339
*p*-menth-1-en-8-ol (MO)	24.944	1692–1704	1701	2.074
Vinyl crotonate	-	-	-	N.D.
Menthol	-	-	-	N.D.
Borneol (MO)	30.458	1692–1706	1698	1.999
Nerolidol (SO)	30.458	2006	1998	0.034
Cinnamaldehyde	-	-	-	N.D.
Total				99.999
monoterpenes				82.61
sesquiterpenes				13.04
others				4.34
Total				99.99

LRI rep—linear retention index reported in the literature, LRI exp—linear retention index from the current analysis, N.D.—not detected; MH—hydrogenated monoterpenes, MO—oxygenated monoterpenes, SH—hydrogenated sesquiterpenes, SO—oxygenated sesquiterpenes.

**Table 3:** The inhibition percentage of *Mentha piperita* L. and *Rosmarinus officinalis* L. essential oils at different concentrations (5, 10, 25, 50, 75, 100 and 200 µg/mL).

Conc. µg/mL	IP (%) M_EO	IP (%) R_EO
5	36.52 ± 0.04 ****	34.25 ± 0.02 ****
10	37.51 ± 0.04 ****	34.55 ± 0.03 ****
25	45.00 ± 0.07 ****	35.14 ± 0.04 ****
50	52.44 ± 0.1 ****	35.66 ± 0.04 ****
75	57.38 ± 0.2 ****	35.83 ± 0.04 ****
100	58.28 ± 0.2 ****	37.21 ± 0.04 ****
200	65.89 ± 0.2 ****	38.56 ± 0.02 ****

**Table 4:** The results of antibacterial activity of *M. piperita* and *R. officinalis* essential oils.

Microbial Strain	Test Compound	IZ (mm)	MIC (µg/mL)	MBC (µg/mL)
*Streptococcus mutans* (+)	M_EO_1	31.67	1.25	1.25
	M_EO_2	24.33	0.625	0.625
	M_EO_3	21.67	0.625	0.625
	R_EO_1	13.00	-	-
	R_EO_2	12.33	-	-
	R_EO_3	8.67	-	-
	Positive control (GM)	19.67	-	-
	Negative control (EtOH)	7.67	-	-
*Streptococcus pyogenes* (+)	M_EO_1	33.33	1.25	1.25
	M_EO_2	31.33	0.625	0.625
	M_EO_3	24.33	0.625	0.625
	R_EO_1	12.67	-	10
	R_EO_2	12.00	-	-
	R_EO_3	9.00	-	-
	Positive control (GM)	20.67	-	-
	Negative control (EtOH)	8.00	-	-
*Staphylococcus aureus* (+)	M_EO_1	18.00	2.5	2.5
	M_EO_2	18.00	1.25	1.25
	M_EO_3	12.00	1.25	1.25
	R_EO_1	20.00	2.5	2.5
	R_EO_2	12.00	-	-
	R_EO_3	11.67	-	-
	Positive control (GM)	20	-	-
	Negative control (EtOH)	7	-	-
*Escherichia coli* (−)	M_EO_1	17.67	2.5	2.5
	M_EO_2	17.33	1.25	1.25
	M_EO_3	15.33	1.25	1.25
	R_EO_1	18.33	2.5	2.5
	R_EO_2	11.67	-	-
	R_EO_3	11.00	-	-
	Positive control (GM)	20.33	-	-
	Negative control (EtOH)	7	-	-
*Pseudomonas aeruginosa* (−)	M_EO_1	11.33	-	-
	M_EO_2	11.00	-	-
	M_EO_3	8.67	-	-
	R_EO_1	20.33	5	5
	R_EO_2	11.33	-	-
	R_EO_3	11.00	-	-
	Positive control (GM)	17.67	-	-
	Negative control (EtOH)	7	-	-

M_EOs_1: 10 μg/mL; M_EOs_2: 5 μL/mL; M_EOs_3: 2.5 μg/mL; R_EOs_1: 10 μg/mL; R_EOs_2: 5 μg/mL; R_EOs_3: 2.5 μg/mL; GM—gentamycin; EtOH—ethanol.

**Table 5:** The results of antifungal activity of *M. piperita* and *R. officinalis* essential oils.

Fungi	Test Compound	IZ (mm)	MIC (µg/mL)	MFC (µg/mL)
*Candida albicans*	M_EO_1	32.33	1.25	1.25
	M_EO_2	30.67	0.625	0.625
	M_EO_3	23.67	0.625	1.25
	R_EO_1	12.67	-	-
	R_EO_2	12.00	-	-
	R_EO_3	8.33	-	-
	Positive control (FCZ)	17.33	-	-
	Negative control (EtOH)	7	-	-
*Candida parapsilosis*	M_EO_1	31.33	1.25	1.25
	M_EO_2	30.33	0.625	0.625
	M_EO_3	23.00	0.625	0.625
	R_EO_1	12.33	-	-
	R_EO_2	12.33	-	-
	R_EO_3	8.00	-	-
	Positive control (FCZ)	18.33	-	-
	Negative control (EtOH)	7	-	-

M_EOs_1: 10 μg/mL; M_EOs_2: 5 μL/mL; M_EOs_3: 2.5 μg/mL; R_EOs_1: 10 μg/mL; R_EOs_2: 5 μg/mL; R_EOs_3: 2.5 μg/mL; FCZ—fluconazole; EtOH—ethanol.

## Data Availability

Not applicable.

## Sample Availability

Samples of the compounds used in the current study are available from the authors.
